# Access, excess, and overdiagnosis: the case for thyroid cancer

**DOI:** 10.1002/cam4.184

**Published:** 2014-01-10

**Authors:** Stephen F Hall, Jonathan Irish, Patti Groome, Rebecca Griffiths

**Affiliations:** 1 Cancer Care and Epidemiology, Queen's Cancer Research Institute, Queen's UniversityKingston, Ontario, Canada; 2 University of TorontoToronto, Ontario, Canada; 3 Cancer Care and Epidemiology, Queen's Cancer Research Institute, Queen's UniversityKingston, Ontario, Canada

**Keywords:** Access, incidence, medical tests, overdiagnosis, thyroid cancer

## Abstract

The incidence of thyroid cancer in women is increasing at an epidemic rate. Numerous studies have proposed that the cause is increasing detection due to availability and use of medical diagnostic ultrasound. Our objective was to compare rates of diagnosis across different health-care regions to rates of diagnostic tests and to features of both health and access of the regional populations. This is a population-based retrospective ecological observational study of 12,959 patients with thyroid cancer between January 1, 2000 and December 31, 2008 in Ontario Canada based on the health-care utilization regions (Local Health Integration Networks) of the province of Ontario Canada. We found that some regions of Ontario had four times the rates of diagnosis of thyroid cancer compared to other regions. The regions with the highest use of discretionary medical tests (pelvic ultrasound, abdominal ultrasound, neck ultrasound, echocardiogram, resting electrocardiogram, cardiac nuclear perfusion tests, and bone scan), highest population density, and better education had the highest rates of thyroid cancer diagnoses. Differences in the rates of the ordering of discretionary diagnostic medical tests, such as diagnostic ultrasound, in different geographic regions of Ontario lead to differences in the rates of diagnosis of thyroid cancer.

## Introduction

According to Canadian Cancer Statistics 1, the incidence of thyroid cancer in women in Canada has been increasing steadily at 7% per year since the mid-2002. Similar increases have been described in other countries 2–5. The causes of the increase have traditionally been assumed to be environmental exposure to atmospheric radiation and iodine insufficiency 6, but recent research on other potential causes includes iatrogenic exposure to radiation 7, reproductive patterns in women 8,9, body mass index (BMI) 10, improved pathology diagnosis 5,11, and increasing detection by diagnostic imaging 2,3,12,13. We have previously reported that in Ontario Canada the increasing incidence of thyroid cancer was associated with the increasing incidence of thyroid cancers under 2 cm 3, and we have demonstrated in Ontario a corresponding increasing use of diagnostic radiology tests on the neck 13. This ecological study pursues the increasing detection theory by comparing rates of diagnosis of differentiated thyroid cancer in the different geographic regions of Ontario to factors of health and health-care access of those regional populations that might influence rates of diagnosis.

## Method

### Study population and data sources

We identified all patients over 18 years of age with a diagnosis of thyroid cancer (ICD 193) in the Ontario Cancer Registry between January 1, 1999 and December 31, 2008 who had a surgical treatment for thyroid cancer between January 1, 2000 and December 31, 2008 (*n* = 13724). The Province of Ontario has a population over 13 million. This cohort was linked at the Institute of Clinical and Evaluative Sciences (ICES) to the Ontario Health Insurance Plan (OHIP) for physician billing codes with dates (surgery, diagnostic radiology, and fine-needle aspiration biopsy [FNAB]) and to the Canadian Institutes of Health Information (CIHI) for hospital procedure codes (surgery) with dates. We compared the OHIP and the CIHI surgical procedure codes and assigned an initial index surgery (hemithyroidectomy, subtotal thyroidectomy, or total thyroidectomy) to each patient. For consistency the date of diagnosis for each patient was the date of the index surgery as FNAB was not performed on all patients. We excluded 769 cases due to discrepancy on the extent of the index surgery, date of index surgery, incomplete information, or incompatible codes. The study population was 12,959 patients treated with a therapeutic surgical procedure for thyroid cancer.

The management of health-care services in the Province of Ontario, Canada, is divided into 14 geographic Local Health Integration Networks (LHINs) based on health-care utilization. With Ontario's diverse geography, the LHINs vary in size, population (over 2 million to 250,000), and urban/rural mix. All but one LHIN has a major teaching hospital with a multidisciplinary cancer treatment center and some LHINs have more than one. For this study the assignment of a patient's LHIN, investigations, and diagnosis (index surgery) was based on patient postal code not the LHIN of the index surgery.

Data on demographics, general health, income, and access to healthcare for the populations of each LHIN were obtained from public sources including websites for Ontario's LHINs 14, ICES 15, the Ontario Hospital Association 16, Cancer Care Ontario 17, and the Ontario Ministry of Health and Long-Term Care. Data were available on the numbers of computed tomography (CT) scanners 18, but unfortunately similar information on diagnostic ultrasound equipment was not. The numbers of operating rooms per LHIN was obtained from Health Systems Accountability and Performance Division, Implementation Branch, Ontario, Ministry of Health and Long-Term Care. We selected 43 factors (Table 1) from these multiple sources that might be associated with the diagnosis of thyroid cancer.

**Table 1:** Factors tested for correlation with the diagnosis of thyroid cancer

*Medical tests as a measure of access to healthcare*	
Rate of NI cardiac stress tests	Rate of holter monitoring
Rate of resting electrocardiogram tests	Rate of echocardiograms
Rate of coronary angiography tests	Rate of ultrasounds – abdomen
Rate of cardiac nuclear perfusion tests	Rate of ultrasounds – pregnancy
Rate of cardiac nuclear wall motion tests	Rate of ultrasounds – pelvic
Rate of sleep studies	Rate of CT scans – neck
Rate of X-rays – chest	Rate of CT scans – thorax
Rate of X-rays – spine	Rate of CT scans – total
Rate of nuclear bone scans	Rate of magnetic resonance imaging (MRI) – total
*General access to healthcare*	
Hip replacement rate	Number of CT scanners
Knee replacement rate	Number of acute care beds
Cataract surgery rate	Number of operating rooms
Rate of family physicians	Cancer surgery wait times
Percentage of population without a regular medical doctor	Participation in OBSP and non-OBSP screening
Percentage of women (age 50–69) who are up-to-date for cancer screening	Percentage of women (age 20–74) who are up-to-date for cancer screening
Percentage of women who are up-to-date for pap tests	
*Education and income*	
Average total income	Proportion of postsecondary graduates (age 25–54)
Proportion of high school graduates (age 25–29)	Percentage of the population with income > $60,000 per year
*Determinants of health*	
Obesity: percentage of population (18+) self-reporting obesity	Smoking: percentage of adults (aged 20+) who are current smokers
Vegetable and fruit intake: percentage of adults (aged 18+) eating vegetables and fruit 5 or more times daily	Physical activity: percentage of adults (18+) who are active or moderately active
Alcohol: percentage of adults (aged 19+) not following the Centre for Addiction and Mental Health (CAMH) low-risk drinking guidelines	
*Miscellaneous*	
Population density (square km)	Percentage of population Over 50
Average cancer patient satisfaction scores for emotional support	

### Analysis

We calculated the mean rates of diagnosis per 100,000, defined as the total number of cases for 9 years for the total population of those years, and compared them by LHIN. The 95% confidence intervals are based on the mean of the rates over the 9 years assuming a Poisson distribution. We then compared the rank order of the LHINs for mean diagnosis rates to available data on the 43 factors by LHIN (Spearman correlation) and report the factors with *r* > 0.50 and *r* < 0.05 with *P* < 0.05. We also compared the numbers of FNABs by a radiologist, rates of diagnostic ultrasound of the neck, and rates of FNAB to the mean rates of diagnosis by LHIN.

## Results

**Figure 1:**
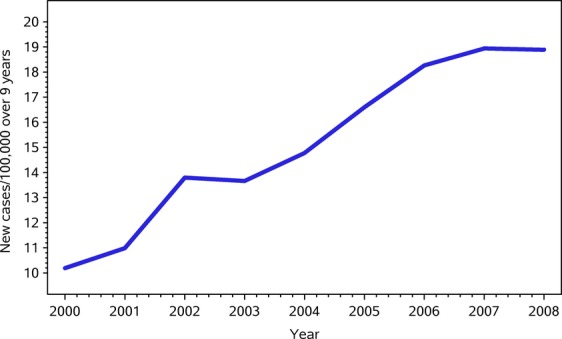
New cases per 100,000 by year in Ontario.

There was overall a 112% increase in the numbers of new cases (893–1890) over the 9 years (Fig. 1) with an increase from 10.19/100,000 to 18.89/100,000 across Ontario.

Eighty percent of patients were female. The average age of females was 47.1 years, 3.5 years younger than males. Papillary (and Follicular) carcinoma accounted for 98.0% of cases with a histological diagnosis (the pathology diagnostic code was missing for 6.3% of cases [*n* = 815]). There were 184 cases of medullary carcinoma and 57 cases of anaplastic/insular carcinoma.

**Figure 2:**
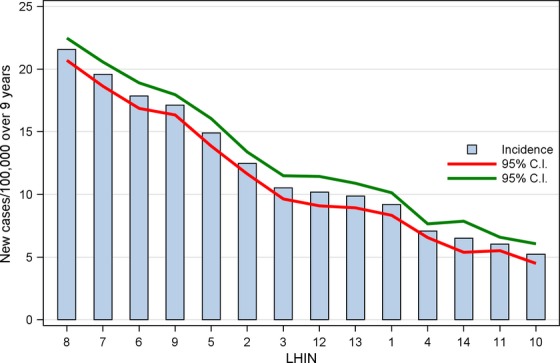
Diagnosis rate (per 100,000) over 9 years by LHINs.

Figure 2 presents the mean diagnosis rate/100,000 of differentiated thyroid cancer over the 9 years by LHIN with 95% confidence intervals. The order of LHINs on Figure 2 is from greatest to smallest diagnosis rate. Each LHIN had a unique average diagnosis rate and the populations in LHINs 7, 8, and 9 have almost four times the mean rate of case diagnosis over the populations in LHINs 10 and 11. Each LHIN had a different rate of change in the numbers of cases per year over the 9 years reflecting differences in practice evolution, but when we examined by 3-year sections there were only small shifts in the rank order of midrate LHINs (Fig. 2) in each of the 3 years (data not included). Age and gender adjustments were not practical due to the small number of cases per year in some LHINs and because we used the rates based on the average over 9 years. We examined the age and gender indirectly standardized incidence ratios for each LHIN compared to the population of all Ontario in 2004, and found that the adjusted ratios reflected the actual rates and did not affect the rank order of the LHINs on Figure 2. We also generated direct gender-adjusted rates (female) by year for Ontario standardized to the 1991 Canadian population and the yearly rates for Ontario were slightly, but not statistically higher, than the standardized rates for Canada. The Canadian Cancer Statistics 2012 report found Ontario had the highest incidence of thyroid cancer compared to all other provinces 1.

Nine of the 43 factors of the LHIN populations or their access to health-care services had a positive correlation with the order of the mean diagnosis rates of thyroid cancer by LHINs (Table 2). The access factors with highest correlations were rate of nonobstetrical pelvic ultrasound and rate of abdominal ultrasound, tests that are not indicated in the workup for thyroid cancer (or a neck mass) and tests that would not uncover serendipitously an asymptomatic thyroid cancer. Factors from the population of each LHIN that correlated were population density and education. Seven factors with negative correlation (*r* < −0.50 and *P* < 0.05) included rates of joint replacement (hip and knee), obesity, smoking, levels of physical activity, levels of addictions, and percent of population over 50 years of age. Although the average income and proportion of the population with income over $60,000 did not correlate statistically, the three LHINs with highest mean rates of diagnosis had the highest average incomes and were three of the four LHINs with the highest proportion of the population with income over $60,000.

**Table 2:** Factors with positive correlations with the diagnosis of thyroid cancer

Factors	*r*_s_	*P*-value
Ultrasound – nonobstetrical pelvis (per 100,000)	0.85	0.0001
Ultrasound – abdomen (per 100,000)	0.74	0.0024
Echocardiogram	0.66	0.0100
Resting electrocardiogram	0.60	0.0233
Nuclear bone scan	0.56	0.0389
Cardiac nuclear perfusion test	0.54	0.0470
Population density (square km)	0.72	0.0035
Proportion of postsecondary graduates (age 25–54)	0.58	0.0307
Proportion of high school graduates (age 25–29)	0.56	0.0389

**Figure 3:**
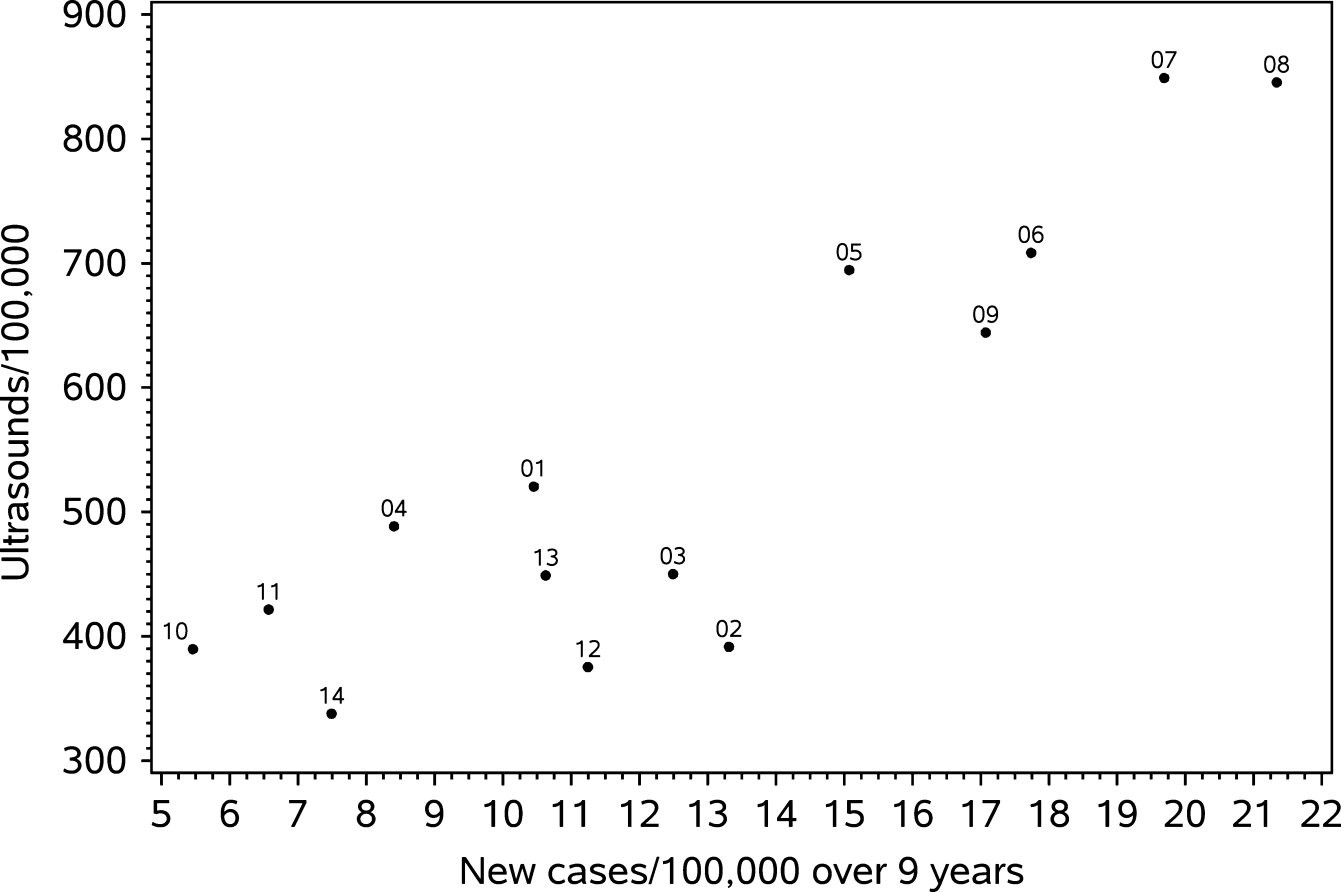
Scatter plot of the number of neck ultrasound examinations (per 100,000) versus diagnosis rate (per 100,000) over 9 years for each LHIN.

The data on the scatter plot (Fig. 3) show the separation of the LHINs into two distinct groups of practice style when comparing the rates of use of neck ultrasound with rates of cancer diagnosis. The populations from regions of Ontario with the higher rates of thyroid cancer had had on average twice as many neck ultrasounds. For this figure, the thyroid cancer cohort was excluded and only one neck ultrasound was counted per person.

Thyroid cancer is increasingly being diagnosed by FNAB often done under ultrasound guidance. The increasing numbers of FNABs per year done by Ontario radiologists essentially matched the increasing rate of cases (*r* = 0.98, *P* < 0.0001), the percent of thyroid cancer patients having FNAB done by a radiologist increased from 44.4 to 68.7 over the 9 years, and the correlation between rates of FNAB and cancer diagnosis by LHIN was 0.89 (*P* = 0.0001).

## Discussion

The objective of this study was to identify factors of access to healthcare or characteristics of the population in different geographic regions that might explain the differences in rates of thyroid cancer diagnosis. There are large differences in rates of use of medical diagnostic ultrasound across the province of Ontario and the regions with the highest use of diagnostic ultrasound (neck, abdomen, and pelvis) also had the highest rates of thyroid cancer diagnosis. These regions also had the highest rates of discretionary cardiac investigations including resting electrocardiograms, echocardiograms, and nuclear cardiac scans. Features of the population of the LHINs with the highest rates of diagnosis were higher education, urban location, and overall health. The negative correlation with joint replacement is consistent with the younger age of the thyroid cancer population. Rates of CT of chest and neck did not correlate, although these tests do on occasion identify asymptomatic thyroid pathology. These findings suggest that differences in access to or use of medical diagnostic tests by more educated, urban, and healthier patient populations are driving the increasing rates of thyroid cancer diagnosis in Ontario. The indications for medical ultrasound imaging for any site as well as other discretionary tests such as resting electrocardiogram, nuclear bone scan, and cardiac perfusion test and why the rates of use are so different between LHINs is beyond the scope of this project, but investigations of these practices could reveal information and processes that could dramatically reduce health-care costs.

This study was possible with the universal healthcare in the Province of Ontario, the legislated collection of data from multiple sources on all patients, and the availability of centralized administrative data sets, and these are the strengths of this study. There are potential weaknesses. First is the potential for ecological fallacy 19. We can conclude that the populations of the different LHINs have different average characteristics and different rates of thyroid cancer diagnoses, but we cannot conclude that the individuals who had specific tests or have specific health characteristics (nonsmokers or better health) have a different rate of diagnosis of thyroid cancer. Further investigation is warranted at the individual level and is possible given the data sources. The second potential weakness is the heterogeneity of the populations of the LHINs. The LHINs were created based on patterns of health-care utilization so reflect referral patterns and major hospitals, but the boundaries do not create homogeneous groups of people. This almost certainly confounded the income results as the LHINs with highest incomes are those with the largest cities, and further investigation based on smaller units such as electoral units and/or the 70 sub-LHINs would contribute to a better understanding of some of the relationships we have identified. The third potential weakness is our use of mean diagnosis rate by LHIN. This is an average rate over the 9 years so does not reflect the different individual rates of increase in cases over the 9 years that occurred within each LHINs. Forth is that we may have slightly underestimated or overestimated the study population. For example, there will have been a small number of patients who despite universal healthcare did not have surgery for FNAB +ve thyroid cancer. These would be balanced by patients with false +ve FNA, who if coded as thyroid cancer in the Ontario Cancer Registry would have been included. Fifth is that our analysis is not age and gender adjusted. This was not practical due to small numbers of cases in some years for some LHINs, was not appropriate when we used the total number of cases over 9 years, and adjusted rates would not be comparable to rates of tests or LHIN population data. When we examined the age and gender indirectly standardized incidence ratios for each LHIN, the rank order did not change. Based on the 2004 census data, there were 2% more females between 20 and 50 years of age in the LHINs with the highest rates of diagnosis of thyroid cancer compared to the other LHINs, but this small difference is unlikely to have created such large differences in rates/100,000 of diagnosis. Sixth, we compared factors on health-care access by LHIN when patients may have had their diagnostic index surgery in another LHIN. Further analysis did show that trans-LHIN migration for index surgery was common in LHINs 5, 6, 7, 8, and 9, but rarely outside those regions and rarely between the higher and lower rate regions (data not included). Having surgery in a different region could be patient choice, referring physician choice, or could be a reflection of resources or expertise within a patient's region. Seventh, we have not provided any insight into the other potential causes of the increase in thyroid cancer as we have no information by LHIN on levels of radioactive fallout, iodine insufficiency, use of hormone replacements, differences in histological interpretation by pathologists, or rates of past iatrogenic radiation. Our finding that obesity rates by LHIN negatively correlated with thyroid cancer rates (−0.59, *P* = 0.027) does not support the hypothesis by Peterson et al. related to BMI noting that these two measures may not be comparable. Eighth, a bias in the correlation between tests and rates is possible due to patients with radiation exposure from excessive diagnostic radiology testing who went on to develop thyroid cancer; however, these case numbers would be small and the major correlations are very strong. Finally, we have used multiple comparisons that allowed us to look at more factors, but increased the risk of chance or false relationships. Using a population-based approach we have identified a number of strong correlations between access, patient characteristics, and the rates of diagnosis of thyroid cancer, and more intensive investigation is warranted.

Our results are similar to an ecological study on thyroid cancer by Sprague et al. 20. The authors examined the incidence of thyroid cancer in Wisconsin between 1980 and 2004 by county and compared incidence to education, income, and health insurance. The incidence of thyroid cancer almost doubled between 1980 and 2004 and increased at 4% per year after 1990. Fifty percent of the increase was attributable to tumors less than or equal to 1 cm in size, that is, likely not palpable and not symptomatic. The increase varied 7.8-fold across the 72 counties and the variation had moderate correlation with median household income, percent of county residents with a college degree, and percent of county residents with health insurance (*r* = 0.41). They concluded that “These results are consistent with the proposed role of improved diagnostic techniques in the increasing incidence. The possible overdiagnosis of thyroid cancer may contribute to unnecessary medical care”. Another ecological study by Roche et al. 21 found a positive correlation between thyroid cancer incidence and socioeconomic status (based on deprivation scores) and between incidence and the percent of residents with health insurance by county based on a study of 15,576 cases in the New Jersey State Cancer Registry from 1979 to 2006 suggesting access as the driver in the incidence of thyroid cancer.

We have demonstrated that the percent of FNABs done by a radiologist had a very strong correlation with the diagnosis of thyroid cancer between 2000 and 2008. In previous work 3 we reported that in Ontario there is an increasing number of patients with tumors less than 2 cm in size, many of which would have been asymptomatic and therefore only found on screening tests such as neck ultrasound. Similarly, Davies 2, Chen 22, and Morris 11 reported that the number of patients with smaller tumors was rising faster than the groups with larger tumors. An ultrasonographer can identify smaller tumors with greater accuracy; thus, the strong correlation is not unexpected and supports the finding of the increasing number of smaller tumors.

Overdiagnosis occurs when a condition is diagnosed that would otherwise not go on to cause symptoms or death, and includes nonprogressive or very slow-growing cancers 23. When rising incidence is associated with stable mortality, as reported for thyroid cancer 20, 23, overdiagnosis is a possible explanation. For overdiagnosis of a medical condition to apply, there must be a reservoir of subclinical cases, a diagnostic (or screening) test, and evidence of nonprogression. There have been numerous autopsy series describing the rates of undetected thyroid cancer and all have reported higher rates than the incidence in the general population^24^–^27^,^27^. For example, Harach 25 found micropapillary cancers in over 30% of adults at autopsy. Numerous clinical studies confirm those findings including Takebe 29 et al. who reported the incidence of micropapillary carcinoma to be 3.5% of females or 1000 times the clinical incidence in Japan based on ultrasound and FNAB. Thyroid cancer does have a reservoir of subclinical disease. The second criterion is a diagnostic event (or screening test) that identifies the condition in the absence of symptoms such as diagnostic ultrasound. In some regions of Ontario, up to 50 patients had a neck ultrasound for every new thyroid cancer diagnosis. The third criterion is evidence of nonprogression and there is increasing evidence to suggest that small thyroid carcinomas may have a benign clinical course. For example, Ito et al. 30,31 have published an observation study of highly selected patients with micropapillary cancers found on ultrasound. Of 350 patients, only 16% of their tumors grew 3 mm or more over the average follow-up of 74 months. Thyroid cancer therefore fits all three criteria for the potential for overdiagnosis. If a substantial proportion of undiagnosed cases are in fact nonprogressive and noting that the Ontario LHINs with highest use of neck ultrasound had 25% more cases than the other LHINs, how many of those 3240 patients, most of which would have been asymptomatic, would have ever been diagnosed with thyroid cancer or progressed to clinical presentation without treatment? Perhaps it is time for clinicians to consider clinically relevant and irrelevant thyroid cancer cases similar to other slow-growing or benign-behaving malignancies including renal cell carcinoma 32–34 and prostate 35.

## Conclusion

The funders of healthcare are facing progressively increasing costs due to the increasing numbers of women diagnosed with thyroid cancer. The rates of diagnosis varied up to four times across the medical geographic regions of Ontario and are strongly related to the variation in rates of use of discretionary medical tests including diagnostic ultrasound. Further research is required to identify reasons for variations in ordering of tests. Thyroid cancer fulfills the criteria for overdiagnosis, and given the increasing caseload thyroid oncologists ought to be considering the clinical relevance of some cases.
